# Platinum-Based Interdigitated Micro-Electrode Arrays for Reagent-Free Detection of Copper

**DOI:** 10.3390/s21103544

**Published:** 2021-05-19

**Authors:** Robert Daly, Tarun Narayan, Han Shao, Alan O’Riordan, Pierre Lovera

**Affiliations:** Nanotechnology Group, Tyndall National Institute, T12 R5CP Cork, Ireland; robert.daly@tyndall.ie (R.D.); tarun.narayan@tyndall.ie (T.N.); han.shao@tyndall.ie (H.S.); alan.oriordan@tyndall.ie (A.O.)

**Keywords:** electrochemical sensors, environmental monitoring, heavy metals, pH control

## Abstract

Water is a precious resource that is under threat from a number of pressures, including, for example, release of toxic compounds, that can have damaging effect on ecology and human health. The current methods of water quality monitoring are based on sample collection and analysis at dedicated laboratories. Recently, electrochemical-based methods have attracted a lot of attention for environmental sensing owing to their versatility, sensitivity and their ease of integration with cost effective, smart and portable readout systems. In the present work, we report on the fabrication and characterization of platinum-based interdigitated microband electrodes arrays, and their application for trace detection of copper. Using square wave voltammetry after acidification with mineral acids, a limit of detection of 0.8 μg/L was achieved. Copper detection was also undertaken on river water samples and compared with standard analytical techniques. The possibility of controlling the pH at the surface of the sensors—thereby avoiding the necessity to add mineral acids—was investigated. By applying potentials to drive the water splitting reaction at one comb of the sensor’s electrode (the protonator), it was possible to lower the pH in the vicinity of the sensing electrode. Detection of standard copper solutions down to 5 μg/L (ppb) using this technique is reported. This reagent free method of detection opens the way for autonomous, in situ monitoring of pollutants in water bodies.

## 1. Introduction

An essential component in human development and survival is access to clean and safe water [1]. Unfortunately, water can be contaminated by a variety of pollutants such as pesticides used in agriculture, antibiotics from both human and animal consumption or heavy metals from manufacturing. Pollution from heavy metals is a serious concern due to their adverse effects on human health [2]. Of these heavy metals, copper is widely distributed in the environment due to its use in a range of anthropogenic processes. Copper can be found in many fertilizers in agriculture [3]. It is also an essential component in the manufacturing of electronics [4] and improper disposal of electronic products can lead to excess copper in the environment. Many pipes in old plumbing systems in Ireland use copper piping. This can lead to copper leaching into the water system [5]. Finally, mining activities or mismanagement of abandoned mines can also be another source of copper run-off [4].

Excess heavy metal pollution in the environment presents a major threat for many ecosystems. It can affect aquatic systems and animals [3]. As these metal ions are persistent and do not break down further in environmental systems [6], they can then bioaccumulate through the food chain, eventually affecting human food produce [4]. While trace amounts of some heavy metals are essential for life [7], these can be detrimental to human health at higher concentrations. This includes copper [8]. For this reason, the World Health Organization (WHO) and the European Union (EU) have set the maximum permissible allowed concentration of copper in drinking water as 2 mg/L^−1^ [9]. Increased levels of copper can lead to various human disorders such as Wilsons Disease [10,11], Alzheimer’s [12], Parkinson’s [13], Prion, Huntington’s disease [14] and disorders such as diabetes and obesity [15]. Other symptoms caused by excess copper include stomach cramps, vomiting and gastrointestinal issues [16]. As a result, environmental monitoring of copper levels is of critical importance for both ecological and public health preservation. Ideally, a monitoring system capable of in situ, real time and sensitive detection could provide early warning pollution alert that could possibly be used to mitigate the impact of pollution events.

Conventional analytical detection methods for heavy metals such as copper include Atomic Absorption Spectroscopy (AAS) [17], Inductively Coupled Plasma Mass Spectrometry (ICP-MS) [18] and Inductively Coupled Plasma Atomic Emission Spectrometry (ICP-AES) [19]. Fluorescence [20], colorimetric [21] and spectroscopic methods such as Raman [22] and Surface Enhanced Raman Scattering [23] have also been used for detection of heavy metals. However, these methods are laboratory-based and require expensive, bulky instrumentation, trained personnel and some require sample pre-treatment making them unsuitable for on-site or in situ testing. In this respect, electrochemical methods have attracted a lot of attention since they can provide a rapid, cost effective and sensitive method for on-site analysis of heavy metals without the need for lab or trained operators. Furthermore, the readout electronic boards can host data analytics protocols and wireless communication technologies enabling smart, autonomous, networked and remote environmental monitoring system [24].

A variety of electrode materials have been employed for detection of copper, see Table 1. These include glassy carbon [3], imprinted polymeric film [8], zeolite/expanded graphite structures [19], gold modified with mercaptoethanesulphonate [25], multi-wall carbon nanotubes-carbon paste [26], screen printed electrodes [2,27], sol-gel Au nanoparticle-carbon paste electrodes [28], quantum dots [29,30], graphene like carbon [30,31], iridium micro disks [32], surfaces modified with chelating agents [16,33] and metal oxides [30,34]. Current electrochemical methods are mainly based on macro sized electrodes. However, sensors based on microscale electrodes have been shown to have enhanced mass transport properties, leading to faster response time with improved sensitivities [35,36]. Interdigitated electrodes in particular have been shown to combine small footprint with sensitive detection towards heavy metals such as, Lead (Pb) [37], Mercury (Hg) [38], Silver (Ag) [39], Zinc (Zn) [40] and Cadmium (Cd) [41]. Interdigitated electrodes based on Sol-Gel-Immobilized-Urease Biosensor [42] and L-Cysteine Functionalized Au [43] have been successfully used for detection of copper, with LOD of 0.31773 and 4.14 × 10^−13^ g/L reported, respectively.

A number of electrochemical techniques can be used for sensing applications. Amongst them, square wave anodic stripping voltammetry (SWASV) has been shown to be particularly suitable for detection of heavy metals. This method involves two steps, the first step being the pre-concentration of the target metal at the surface of the electrode. This step is then followed by oxidative stripping of the metal from the surface by sweeping the potential through the metal’s oxidation potential. Detection limits of copper in the μg·L^−1^ range can be readily achieved using this method.

An important consideration for the electrochemical detection of heavy metals is the sample pH. Indeed, the pH can affect the availability of metal ions for electrochemical processes. Generally, electrochemical methods are performed by adding of acids in order to chemically adjust the pH to that needed for the measurements. This means that some sample preparation is required for the analysis. This cannot be easily integrated into portable devices, meaning true in situ measurements are impossible.

The approach investigated here is to negate the need for chemical reagent addition completely by incorporating on-chip local pH control. This is achieved by the electrolytic decomposition of water to form H^+^ ions at one interdigitated electrode comb. The sensors are comprised of two interdigitated arrays of platinum (Pt) electrodes. Electrode microbands are spaced 2 μm apart. One side, or comb, of the array is used as the working electrode and carries out the SWASV as it would normally. The opposite side of the comb is held at a constant potential to adjust the pH to the required pH. This method has been shown to work for the detection of free chlorine [44], silver [45] and has also been used for the elimination of oxygen interference in the detection of monochloramine [46]. Aside from the possibility of locally changing the pH, the sensors described herein have the potential to be integrated into a portable, autonomous system for remote, multiplexed monitoring of copper levels.

## 2. Materials and Methods

### 2.1. Chemicals and Solutions

Copper sulphate (CuSO_4_), Nitric acid (HNO_3_), Sodium Chloride (NaCl), Phosphate Buffer Saline (PBS) and Ferrocenecarboxylic Acid (FCA) were obtained from Sigma Aldrich, (Wicklow, Ireland). All aqueous solutions were prepared using ultra-pure Milli-Q water (18.2 MΩ·cm^−1^, Milli-Q, Dublin, Ireland). Standard solutions of copper were made up using 10 mM NaCl and diluted as needed from a stock solution. These standards were adjusted to pH 2 by addition of 1 M HNO_3_ added dropwise until the desired pH was achieved. All chemicals were used as received without any further purification.

### 2.2. Instrumentation

All electrochemical measurements were undertaken using a portable CH Instrument 1220C bi-potentiostat (CH Instruments, Inc Austin, Tx) three-electrode configuration was used for copper detection using platinum interdigitated microband arrays (IDA) as the working (or sensing) electrode, and gold and platinum on-chip counter electrode and pseudo reference electrode, respectively. In some instances, an Ag/AgCl reference electrode was used—using this electrode was found to shift the voltammograms by ~ +250 mV and all potentials (deposition potential, acquisition potential window) were shifted accordingly. An additional platinum interdigitated microband array (the protonator) was introduced and used in a four-electrode configuration for electrochemical pH adjustment.

### 2.3. Sensor Design and Fabrication

**Figure 1:**
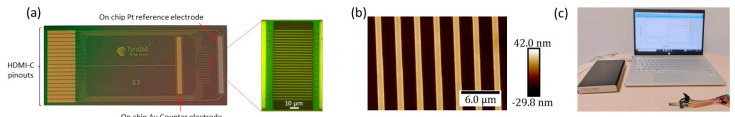
(**a**) optical micrograph of the HDMI-C sensor chip, showing the Au counter electrode, the 8 Pt working electrodes and the Pt reference electrode; with higher magnification of one of the working electrodes; (**b**) Atomic Force microscope image of the working electrode. The interdigitated microbands are 1 μm wide with 2 μm gaps; (**c**) Experimental setup comprising sensor chip, connector, portable bipotentiostat and laptop.

The fabricated sensor chips were 20 mm × 8.5 mm with 19 connection pinouts on the left-hand side. The design was compatible with HDMI-C connector, permitting facile electrical connection to external electronics. Sensor chip fabrication was similar to the one described by Wahl et al. [47] and was based on lithographic processes commonly used in microelectronic foundries, and are therefore compatible with mass manufacturing. The sensors were fabricated on four-inch silicon wafer substrates with a 300 nm layer of thermally grown silicon dioxide. Firstly, the working electrodes were patterned using photolithography and thermal evaporation (50 nm of Pt, with 10 nm of Ti adhesion layer) followed by lift off. A second optical lithographic and metal deposition process (Ti 10 nm/Au 100 nm) was undertaken to define HDMI pin-out, interconnection tracks, as well as the on-chip counter electrode (500 μm wide × 6 mm long). A third similar step was used to define the Pt (Ti 10 nm/Pt 100 nm) on-chip reference electrode (500 μm wide × 6 mm long). The distance between the WE and RE is 1 mm. Such a small distance allows ohmic drop issues to be mitigated [48]. Finally, 500 nm of PECVD SiN was blanket deposited on the whole wafer, and openings over the working/counter/reference electrodes and electrical contacts defined by lithography and dry etching. An optical image of a fabricated chip can be seen in Figure 1a. A die contained 8 individual sensors, allowing for multiplexed measurements if needed. Each sensor comprised two interdigitated arrays (IDA); the protonator IDA comprised 14 tines while the working IDA comprised 13 tines. Each tine was 1 μm wide and 45 μm long, and the gap between tines was 2 μm, see high magnification in Figure 1a,b. Prior to the experiments, each chip was inspected using optical microscopy to identify any obvious defects or faults. The chips were washed with isopropanol and deionised water to remove any residual contaminants from the electrode surface. The overall experimental setup comprised the sensors chips, the HDMI-C connector, a portable bipotentiostat and a laptop (see Figure 1c) and could easily be brought on the field for on-site measurements.

### 2.4. Optical and Surface Morphology Characterization

Optical micrographs were acquired using a calibrated microscope (Axioskop II, Carl Zeiss Ltd Cambridge, UK.) equipped with a charge-coupled detector camera (CCD; DEI-750, Optronics, Wembley, UK). The surface morphology of the sensors was studied using a Bruker Nanoscope dimension icon atomic force microscope in tapping mode. The surface morphology and compositional analysis of the electrodeposited copper samples were performed using a field emission scanning electron microscope (FEI QUANTA 650 HRSEM, FEI UK Ltd) with energy dispersive X-ray spectroscopy (EDX Oxford Instruments INCA energy system, Oxford, UK).

### 2.5. Copper Detection Using Chemical pH Adjustment

**Figure 2:**
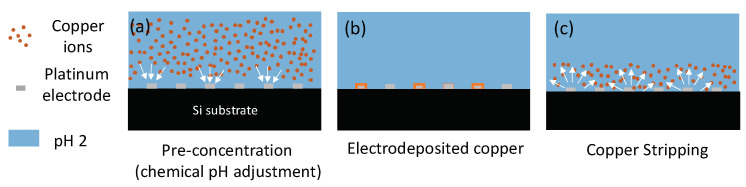
Schematic of electrochemical detection of copper. (**a**) pre-concentration at the surface of the working electrode; (**b**) electrodeposition of copper at the surface of the electrode and (**c**) stripping of deposited copper back in solution.

The electrochemical detection of copper was carried out in a multi-step process, see Figure 2. First, a 50 μL aliquot of the solution to analyse, adjusted to pH 2 with nitric acid, was pipetted onto the active area of the sensor chip. The next step was the electrodeposition of copper ions present in solution onto the surface of the platinum working electrode. This was achieved by applying a potential of −0.65 V to the Pt working electrode. Following this, square wave voltammetry was used to strip the as deposited copper back into the solution. This resulted in an oxidation peak with a peak height proportional to the concentration of copper present in solution.

### 2.6. River Water Sample Collection

**Figure 3:**
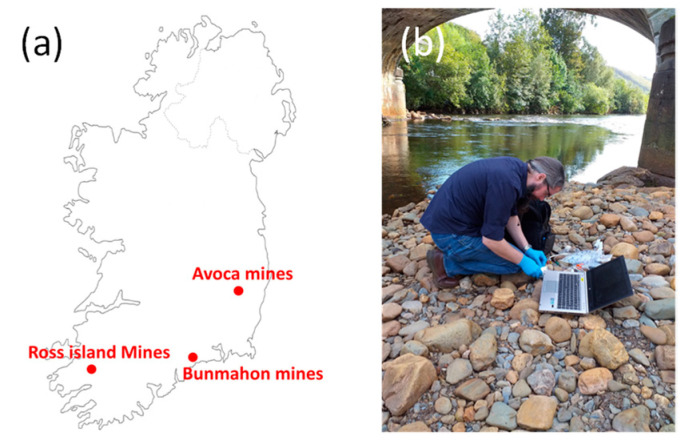
(**a**) Map of Ireland showing the sampling/measurement locations; (**b**) measurement being carried on-site at Avoca river, co Wicklow.

Analysis of water collected at Avoca (Co. Wicklow), Ross island (Co. Kerry) and Bunmahon (Co. Waterford) was undertaken, see Figure 3a. These sites were selected as they were expected to have high copper levels due to their proximity to disused copper mines [49] and could also be easily and safely accessed. Water samples were analysed on-site (i.e., by taking a sample of water, and carrying out the analysis on the side of the water body), see Figure 3b. Grab samples were also brought back to the laboratory for further analysis and sent for ICP-MS analysis by an accredited laboratory (Environmental Laboratory Services, Cork, Ireland).

## 3. Results and Discussion

### 3.1. Electrode Array Characterisation

**Figure 4:**
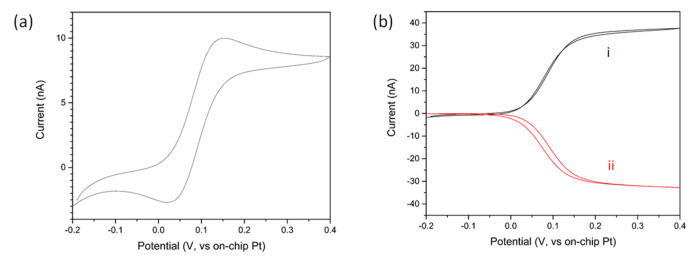
(**a**) Typical cyclic voltammogram in 1 mM FCA in 10 mM PBS measured at a platinum interdigitated electrode comb; (**b**) CVs at the generator and collector IDAs. The generator IDA (i) was cycled between −0.2 V and 0.4 V while the collector IDA (ii) was held at −0.1 V.

Cyclic Voltammetry (CV) was carried out in 1 mM FCA in 10 mM PBS to characterize the electrochemical functionality of the sensors. As can be seen in Figure 4a, the CV has a typical duck shape indicative of diffusion limited behaviour commonly observed at macroelectrodes. This arose as a result of the overlapping of radial diffusion profiles surrounding individual microband of an IDA, thus resulting in an overall time-dependent diffusion limited behaviour [50]. The interdigitated setup was also tested with FCA in a collector generator mode, see Figure 4b. In this approach, one comb of the electrode (the generator) was cycled as above while the other comb (the collector) was held at −0.1 V. As in the previous case, the FCA molecules begin to oxidize to FCA^+^ at the generator for potentials above 0 V. The oxidized species then diffused above the collector where they were reduced back to FCA. As a result of this process, the CV exhibited a steady state profile with higher measured currents (40 nA vs. 9 nA). The collection efficiency of the sensor, defined as the ratio of the collector to the generator currents, was determined to be ∼84%. This suggests that for electrochemical pH adjustment (*vide infra*), 84% of protons generated at the protonator, would diffuse over the working IDA. 

### 3.2. Detection of Cu in Standard Solutions Using Chemical pH Adjustment

**Figure 5:**
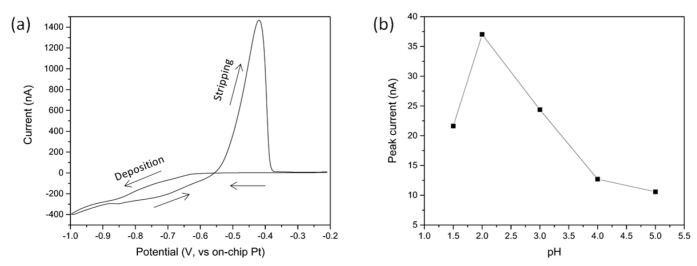
(**a**) Cyclic Voltammogramm of copper electrodeposition onto the Pt microbands (10 mM CuSO_4_, pH 2); (**b**) Influence of the pH of the solution on measured stripping current (100 μg/L CuSO_4_ solution, 300 s deposition at −0.65 V). The line is a guide to the eye only.

Figure 5a shows the cyclic voltammogram for copper deposition from a 10 mM aqueous solution of CuSO_4_ at pH 2. An anodic current corresponding to the reduction of copper ion to copper metal could be seen starting at a potential of approximatively −0.65 V and continuing at lower potential. On the reverse sweep, the deposition continued up to −0.55 V, indicating that the growth of copper is more kinetically favoured on existing Cu nucleation sites. The return sweep also showed a sharp peak at ~ −0.40 V corresponding to the stripping of the copper that was deposited. For the following experiments, a deposition potential of −0.65 V was applied to the working electrode for 300 s (−0.4 V when Ag/AgCl reference electrode was used). Using lower deposition potentials could result in higher amount of copper being deposited but, at low concentration, could also potentially increase the interference from hydrogen-evolution effects—a process particularly efficient at Pt electrodes. SEM and EDX analysis confirmed that the copper deposition was stable on the Pt electrode, see Figure S1. The deposited copper was then stripped from the surface using square wave voltammetry resulting in a peak at approximatively −0.4 V. The following SWV conditions were used: starting potential −0.7 V, frequency 25 Hz, increment potential 0.004 V and amplitude 0.05 V. A second SWV was carried out right after the first one to ensure that all the copper had been removed from the surface of the working electrode.

The influence of the solution pH on the peak stripping current response was studied by varying solution pH from 5 to 1 (100 μg/L CuSO_4_ solution, deposition at −0.65 V for 300 s), see Figure 5b. Decreasing the pH led to a change in the measured stripping current with the maximum stripping peak current found at pH 2. This optimal pH value was thus selected as the pH of choice for further experiments.

**Figure 6:**
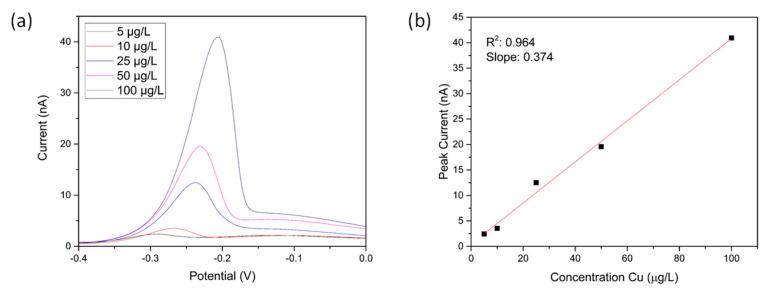
(**a**) Square Wave Voltammograms of Cu solutions at different concentrations (adjusted to pH 2 with acid, deposition for 300 s at −0.4 V); (**b**) Corresponding calibration line.

Square wave stripping voltammetry (see Section 2.5) was used to measure the levels of copper of serial dilutions of copper sulphate solutions adjusted to pH 2. Figure 6a shows the stripping square wave voltammograms obtained for Cu concentrations ranging from 5–100 µg/L. The peak at approximately −0.25 V corresponds to the copper metal being oxidised and stripped from the surface of the electrode. As expected, the intensity of the peaks increased with increasing concentrations of copper in the solution. Concentrations down to 5 μg/L could be readily detected with the experimental conditions chosen. Figure 6b shows the peak intensity as a function of Cu concentration. From this calibration plot, the linear equation i(nA) = 0.85 + 0.37 C_Cu (μg/L)_ with the correlation coefficient of 0.96 was obtained. The limit of detection (LOD) was calculated using Equation (1):

LOD = 3 × SD/S(1)

where SD is the standard deviation of the blank (measured to be 0.1 nA) and S the sensitivity of the electrode, defined as the slope of the calibration line. A LOD of 0.8 μg/L was obtained. It should be noted that lower limit of detections could be achieved with longer deposition times or by enhancing mass transport with a microfluidic setup, but this was not investigated further in the present work as the reported LOD was well below the maximum permissible allowed concentration required by legislation. Additionally, the technique used for benchmarking was limited to 3 μg/L (*vide infra*).

### 3.3. Detection of Cu in Real Samples Using Chemical pH Adjustment

**Figure 7:**
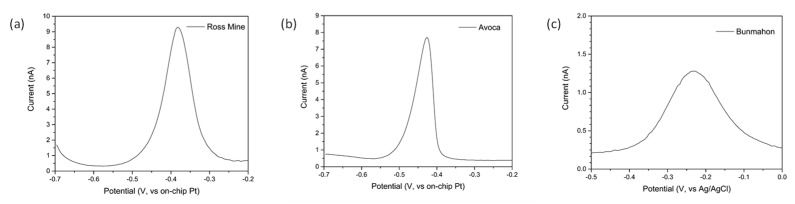
Square wave voltammograms of samples collected from (**a**) Ross mine; (**b**) Avoca and (**c**) Bunmahon (an Ag/AgCl reference electrode was used in this instance) sites. Solutions were adjusted to pH 2 on-site using mineral acid.

In order to test our sensors with more complex matrixes, water samples were collected from sites where measurable levels of copper were expected (see Section 2.6). Samples were grabbed from the water body and acidified to pH 2 on-site using nitric acid. While the collected samples did not show significant turbidity, they were rested for 5 min to allow bigger suspended particulates to fall at the bottom of the tube. Only water from the top of the test tube was used. Figure 7 below shows the stripping square wave voltammograms obtained after 300 s deposition at −0.65 V for the different sites. The measured peak currents (peak to baseline) were 7.7, 9.3 and 1.1 nA for Avoca, Ross mine and Bunmahon, respectively. Using the calibration line established previously, this corresponded to copper concentrations of 17, 20 and 1 ppb, respectively.

These water samples were also sent for ICP-MS analysis in an accredited laboratory and concentrations reported were 22, 27 and < 3 ppb, respectively, see Table 2. Some discrepancy exists between the values measured using the present electrochemical sensors and those obtained with the commercial ICP-MS analysis. These could be due to the electrochemical method adopted here, where sensors detect dissolved free copper only and not copper ions that are chelated to natural organic ligands or copper in suspended solids form such as particulates or colloids—thus underreporting the total copper.

### 3.4. Detection of Cu in Standard Solutions Using Electrochemical pH Adjustment

- Electrochemical pH control

Figure 5 shows that the detection of Cu is more sensitive when the solution is at pH 2. However, typical river samples have a pH around 7. As a result, direct acidification of the sample using mineral acids is generally used for copper sensing. In the present work, the possibility to locally change the pH using electrochemical methods was investigated in order to eliminate the requirement of sample acidification prior to the analysis. The method takes advantage of the efficient catalytic properties of platinum towards the electrochemical water splitting reaction described by Equation (2):

(2) $$2H_{2}O\to 4e^{-}+4\;H^{+}+O_{2}$$

To change the pH at the surface of the sensing electrode, the protonator electrode was biased at potentials inducing reaction (2). The H^+^ ions generated during this chemical reaction diffuse in the volume above the overall microelectrode (sensing electrode and protonator), resulting in a local decrease in the pH at the sensors, enabling in situ pH control.

**Figure 8:**
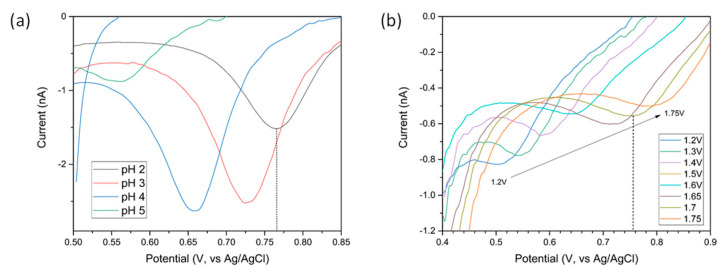
(**a**) Reverse sweep of cyclic voltammograms recorded in a 100 µg/L Cu solution acidified to pH 2 to 5. The AuO/Au reduction peak occurs at 0.76 V at pH 2; (**b**) Selected regions of the cycling voltammogram of a 100 µg/L Cu at pH 7 showing the gold oxide reduction peak when applying potentials 1.2–1.75 V to the protonator. The dashed line shows the location of the gold oxide reduction peak maximum when 1.7 V was applied to the protonator occurring at the same position as when the solution was acidified to pH 2 with acid.

To confirm the electrochemical pH control capabilities of the sensor chips, Au was electrochemically deposited on the sensing electrode. Cyclic voltammograms of 100 µg/L Cu solutions at pH 2 to 5 in the potential range from 0.3–1.2 V of this modified electrode was undertaken. The peaks observed in Figure 8a between 0.55 V and 0.80 V correspond to the reduction of the AuO formed on the forward sweep [51] and occur at 0.76 V at pH 2. pH of the solution can therefore be measured using the potential of the AuO/Au reduction peak as a metric. To test for electrochemical pH control, cyclic voltammograms of the Cu solution at pH 7 was acquired while biasing the protonator at potential inducing the water splitting reaction. Figure 8b shows the AuO reduction peak as a function of the potential applied to the protonator. The AuO/Au reduction peaks can be seen to occur at 0.76 V—indicative of a local pH of 2—when the potential applied to the protonator was set at 1.7 V.

- Detection of Cu in standard solutions using electrochemical pH adjustment

**Figure 9:**
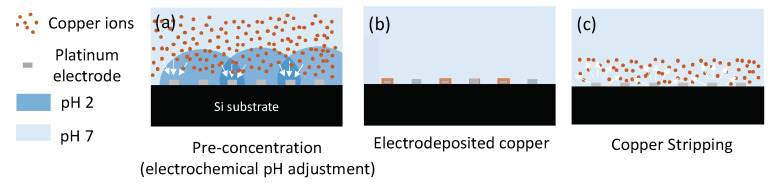
Schematic of detection of copper using electrochemical pH adjustment. (**a**) pre-concentration at the surface of the working electrode when applying 1.7 V at the protonator; (**b**) electrodeposition of copper at the surface of the electrode and (**c**)stripping of deposited copper back in solution.

To demonstrate reagent free detection of copper, the electrochemical pH control was tested on samples spiked with different concentrations of Cu. The deposition and stripping steps are depicted in Figure 9.

**Figure 10:**
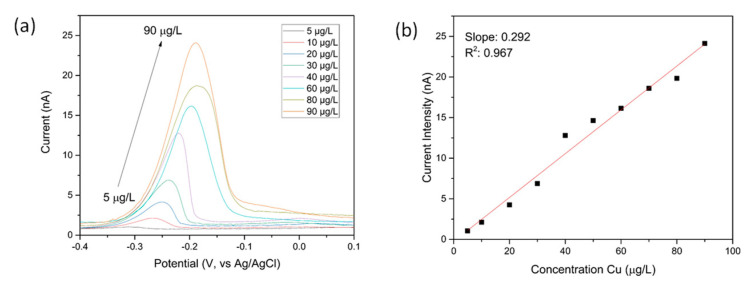
(**a**) Square wave stripping voltammograms of copper solutions acquired using electrochemical pH control. The measurements were done in copper solutions at pH 7 with 1.7 V applied at protonator IDA during deposition; (**b**) Corresponding calibration line.

The protonator was biased at 1.7 V in order to obtain pH 2 at the surface of the sensing comb. The sensing comb was set at −0.4 V as previously in order to reduce the copper ions at the surface of the electrode. The square wave voltammograms of copper solutions at various concentrations can be found in Figure 10a. As in the case of chemical pH adjustment, the intensity of the stripping peak varies linearly with the concentration of the target analyte. Figure 10b shows the corresponding calibration line between 5 and 90 μg/L. A slope of 0.29 nA/(μg/L) was obtained in this case, suggesting the electrochemical pH control method was slightly less sensitive than the chemical pH control one. This might arise as a result of interference from dissolved oxygen also generated in the water splitting reaction.

## 4. Conclusions and Outlook

In summary, we have successfully fabricated platinum interdigitated microband array (IDA) on silicon substrates for trace detection of copper in water. The sensor chips comprised eight individual sensors, allowing for multiplexed sensing, and their fabrication is compatible with mass manufacturing processes. Using square wave anodic stripping voltammetry and addition of mineral acids, the sensors exhibited high sensitivity towards detection of copper ions, with a limit of detection of 0.8 μg/L achieved. These sensors were also used for on-site testing and concentrations of 17, 20 and 1 µg/L were measured at Avoca, Ross and Bunmahon sites. ICP-MS analysis of these samples gave values of 22, 27 and < 3 µg/L, respectively, and the discrepancies with the present sensors was attributed to nature of copper being detected (copper ions for electrochemical sensors vs. total copper for ICP-MS). Detection of copper was also shown to be possible without addition of acids by locally changing the pH using electrochemical processes at the second comb of the IDA. Future work will now focus on developing and optimizing the electrochemical pH control method for analysis in the field.

## Figures and Tables

**Table 1:** Electrochemical sensors for copper detection.

Electrode Type	Linear Range (g/L)	LOD (g/L)	Ref.
Screen Printed Electrodes	0.63546–9.5319	8.9 × 10^−8^	[2]
Glassy Carbon	0.0317–5.084	0.02796	[3]
Imprinted Polymeric Film	5.72 ×10^−8^–9.53 × 10^−6^	1.72 × 10^−7^	[8]
Surfaces Modified with Chelating Agents	6.35 × 10^−8^–0.006355	5.66 × 10^−7^	[16]
Zeolite/Expanded Graphite Structures	NA	1.42 × 10^−7^	[19]
Gold Modified with Mercaptoethanesulphonate (MES)	1 × 10^−6^–8 × 10^−5^	1 × 10^−6^	[25]
Multi-wall Carbon Nanotubes-Carbon Paste	0.063546–6.3546	0.050201	[26]
Sol-gel Au Nanoparticle-Carbon Paste Electrodes	0.0273525–0.63546	0.025418	[28]
Quantum Dots	6.35 × 10^−7^–6.99 × 10^−5^	3.81 × 10^−7^	[29]
Graphene like Carbon	NA	1.46 × 10^−8^	[30]
Iridium Micro Disks	2 × 10^−5^–0.0001	5 × 10^−6^	[32]
Metal Oxides	1.27 × 10^−7^–6.35 × 10^−6^	6.86 × 10^−8^	[34]
Sol-Gel-Immobilized-Urease Biosensor (IDE)	0.063546–0.63546	0.31773	[42]
L-Cysteine Functionalized Au (IDE)	3.18 × 10^−13^–3.18 × 10^−8^	4.14 × 10^−13^	[43]
Platinum IDE	5 × 10^−6^–100 × 10^−6^	0.8 × 10^−6^	Present work

**Table 2:** Comparison of results obtained with ICP-MS and electrochemical sensors.

Sites	ICP-MS (µg/L)	Pt IDA Sensor (µg/L)
Avoca	22	17
Ross Mines	27	20
Bunmahon	< 3	1

## Data Availability

Data is contained within the article.

## Supplementary Materials

The following are available online at https://www.mdpi.com/article/10.3390/s21103544/s1, Figure S1: (**a**) SEM micrograph of the sensor after deposition of Cu from a 100µg/L solution (prior to stripping); The microbands on the left and on the right are part of the sensing comb while the microband in the middle is part of the protonator (**b**), EDX spectrum of the sample, showing Cu peaks at 1 and 8 keV; EDX mapping analysis showing (**c**) Cu and (**d**) Pt contributions.

## References

[1] World Health Organization (WHO) (2018). Developing Drinking Water Quality Regulations and Standards.

[2] Kanyong P., Rawlinson S., Davis J. (2016). Gold nanoparticle modified screen-printed carbon arrays for the simultaneous electrochemical analysis of lead and copper in tap water. Microchim. Acta.

[3] Mourya A., Sinha S., Mazumdar B.. (2019). Glassy carbon electrode modified with blast furnace slag for electrochemical investigation of Cu^2+^ and Pb^2+^ metal ions. Microchem. J..

[4] Wu T., Xu T., Ma Z. (2015). Sensitive electrochemical detection of copper ions based on the copper(II) ion assisted etching of Au@Ag nanoparticles. Analyst.

[5] World Health Organization (WHO) (2008). Guidelines for Drinking Water Quality.

[6] Chen Z., Li L., Mu X., Zhao H., Guo L. (2011). Electrochemical aptasensor for detection of copper based on a reagentless signal-on architecture and amplification by gold nanoparticles. Talanta.

[7] Ramdass V., Sathish V., Babu E., Velayudham M., Thanasekaran P., Rajagopal S. (2017). Recent developments on optical and electrochemical sensing of copper(II) ion based on transition metal complexes. Coord. Chem. Rev..

[8] Di Masi S., Pennetta A., Guerreiro A., Canfarotta F., De Benedetto G.E., Malitesta C. (2020). Sensor based on electrosynthesised imprinted polymeric film for rapid and trace detection of copper(II) ions. Sens. Actuators B Chem..

[9] EU/WHO (2020). Directive (eu) 2020/2184 of the European Parliament and of the Council of 16 December 2020 on the Quality of Water Intended for Human Consumption.

[10] Lutsenko S. (2008). Atp7b−/− mice as a model for studies of Wilson’s disease. Biochem. Soc. Trans..

[11] Mulligan C., Bronstein J.M. (2020). Wilson Disease an Overview and Approach to Management. Neurol. Clin..

[12] Kepp K.P. (2012). Bioinorganic Chemistry of Alzheimer’s Disease. Chem. Rev..

[13] Gangania M., Batra J., Kushwaha S., Agarwal R. (2017). Role of Iron and Copper in the Pathogenesis of Parkinson’s Disease. Indian J. Clin. Biochem. IJCB.

[14] Xiao G., Fan Q., Wang X., Zhou B. (2013). Huntington disease arises from a combinatory toxicity of polyglutamine and copper binding. Proceedings of the National Academy of Sciences of the United States of America.

[15] Nielsen T., Jessen N., Jørgensen J.O.L., Møller N., Lund S. (2014). Dissecting adipose tissue lipolysis: Molecular regulation and implications for metabolic disease. J. Mol. Endocrinol..

[16] Kaur I., Sharma M., Kaur S., Kaur A. (2020). Ultra-sensitive electrochemical sensors based on self-assembled chelating dithiol on gold electrode for trace level detection of copper(II) ions. Sens. Actuators B-Chem..

[17] Álvarez M., Gutiérrez E., Rodríguez A., Sanromán M.Á., Deive F. (2014). Environmentally Benign Sequential Extraction of Heavy Metals from Marine Sediments. Ind. Eng. Chem. Res..

[18] Dai B., Cao M., Fang G., Liu B., Dong X., Pan M., Wang S. (2012). Schiff base-chitosan grafted multiwalled carbon nanotubes as a novel solid-phase extraction adsorbent for determination of heavy metal by ICP-MS. J. Hazard. Mat..

[19] Ma L., Zhang X., Ikram M., Ullah M., Wu H., Shi K. (2020). Controllable synthesis of an intercalated ZIF-67/EG structure for the detection of ultratrace Cd^2+^, Cu^2+^, Hg^2+^ and Pb^2+^ ions. Chem. Eng. J..

[20] Korzec M., Senkala S., Rzycka-Korzec R., Kotowicz S., Schab-Balcerzak E., Polanski J. (2019). A highly selective and sensitive sensor with imine and phenyl-ethynyl-phenyl units for the visual and fluorescent detection of copper in water. J. Photochem. Photobiol. A-Chem..

[21] Chaiyo S., Apiluk A., Siangproh W., Chailapakul O. (2016). High sensitivity and specificity simultaneous determination of lead, cadmium and copper using μPAD with dual electrochemical and colorimetric detection. Sens. Actuators B Chem..

[22] Liu Y., Wu Y., Guo X., Wen Y., Yang H. (2019). Rapid and selective detection of trace Cu^2+^ by accumulation- reaction-based Raman spectroscopy. Sens. Actuators B-Chem..

[23] Dugandzic V., Kupfer S., Jahn M., Henkel T., Weber K., Cialla-May D., Popp J. (2019). A SERS-based molecular sensor for selective detection and quantification of copper(II) ions. Sens. Actuators B-Chem..

[24] Murphy A., Seymour I., Rohan J., O’Riordan A., O’Connell I. (2021). Portable data acquisition system for nano and ultra micro scale electrochemical sensors. IEEE Sens. J..

[25] Herzog G., Arrigan D. (2003). Application of Disorganized Monolayer Films on Gold Electrodes to the Prevention of Surfactant Inhibition of the Voltammetric Detection of Trace Metals via Anodic Stripping of Underpotential Deposits:  Detection of Copper. Anal. Chem..

[26] Ganjali M., Aghabalazadeh S., Khoobi M., Ramazani A., Foroumadi A., Shafiee A., Norouzi P. (2010). Nanocomposite Based Carbon Paste Electrode for Selective Analysis of Copper. Int. J. Electrochem. Sci..

[27] Bernalte E., Arevalo S., Perez-Taborda J., Wenk J., Estrela P., Avila A., Di Lorenzo M. (2020). Rapid and on-site simultaneous electrochemical detection of copper, lead and mercury in the Amazon river. Sens. Actuators B-Chem..

[28] Mashhadizadeh M.H., Talemi R.P. (2011). Used gold nano-particles as an on/off switch for response of a potentiometric sensor to Al(III) or Cu(II) metal ions. Anal. Chim. Acta.

[29] Dong Y., Wang R., Li G., Chen C., Chi Y., Chen G. (2012). Polyamine-Functionalized Carbon Quantum Dots as Fluorescent Probes for Selective and Sensitive Detection of Copper Ions. Anal. Chem..

[30] Cui L., Wu J., Ju H. (2015). Electrochemical sensing of heavy metal ions with inorganic, organic and bio-materials. Biosens. Bioelectron..

[31] She X., Xu H., Xu Y., Yan J., Xia J., Xu L., Song Y., Jiang Y., Zhang Q., Li H. (2014). Exfoliated graphene-like carbon nitride in organic solvents: Enhanced photocatalytic activity and highly selective and sensitive sensor for the detection of trace amounts of Cu^2+^. J. Mater. Chem. A.

[32] Nolan M., Kounaves S. (1999). Microfabricated Array of Iridium Microdisks as a Substrate for Direct Determination of Cu^2+^ or Hg^2+^ Using Square-Wave Anodic Stripping Voltammetry. Anal. Chem..

[33] Lachowicz J., Depiano G., Zanda D., Piludu M., Sanjust E., Monduzzi M., Salis A. (2019). Adsorption of Cu^2+^ and Zn^2+^ on SBA-15 mesoporous silica functionalized with triethylenetetramine chelating agent. J. Environ. Chem. Eng..

[34] Lv J., Zhang C., Wang S., Li M., Guo W. (2021). MOF-derived porous ZnO-Co_3_O_4_ nanocages as peroxidase mimics for colorimetric detection of copper(ii) ions in serum. Analyst.

[35] Berduque A., Lanyon Y.H., Beni V., Herzog G., Watson Y.E., Rodgers K., Stam F., Alderman J., Arrigan D.W.M. (2007). Voltammetric characterisation of silicon-based microelectrode arrays and their application to mercury-free stripping voltammetry of copper ions. Talanta.

[36] Moujahid W., Eichelmann-Daly P., Strutwolf J., Ogurtsov V.I., Herzog G., Arrigan D.W.M. (2011). Microelectrochemical Systems on Silicon Chips for the Detection of Pollutants in Seawater. Electroanalysis.

[37] Cui H., Xiong X., Gao B., Chen Z., Luo Y., He F., Deng S., Chen L. (2016). A Novel Impedimetric Biosensor for Detection of Lead (II) with Low-cost Interdigitated Electrodes Made on PCB. Electroanalysis.

[38] Tekaya N., Saiapina O., Ben Ouada H., Lagarde F., Ben Ouada H., Jaffrezic-Renault N. (2013). Ultra-sensitive conductometric detection of heavy metals based on inhibition of alkaline phosphatase activity from Arthrospira platensis. Bioelectrochemistry.

[39] Soldatkin O., Kucherenko I., Pyeshkova V., Kukla A., Jaffrezic-Renault N., El’skaya A., Dzyadevych S., Soldatkin A. (2012). Novel conductometric biosensor based on three-enzyme system for selective determination of heavy metal ions. Bioelectrochemistry.

[40] Chouteau C., Dzyadevych S., Durrieu C., Chovelon J. (2005). A bi-enzymatic whole cell conductometric biosensor for heavy metal ions and pesticides detection in water samples. Biosens. Bioelectron..

[41] Chouteau C., Dzyadevych S., Chovelon J., Durrieu C. (2004). Development of novel conductometric biosensors based on immobilised whole cell Chlorella vulgaris microalgae. Biosens. Bioelectron..

[42] Ilangovan R., Daniel D., Krastanov A., Zachariah C., Elizabeth R. (2006). Enzyme based Biosensor for Heavy Metal Ions Determination. Biotechnol. Biotechnol. Equip..

[43] Qi H.C., Zhao M.Q., Liang H.G., Wu A.N., Huang Z.F., Hu A.M., Wang J., Lu Y.C., Zhang J. (2019). Rapid detection of trace Cu^2+^ using an l-cysteine based interdigitated electrode sensor integrated with AC electrokinetic enrichment. Electrophoresis.

[44] Seymour I., O’Sullivan B., Lovera P., Rohan J., O’Riordan A. (2020). Electrochemical detection of free-chlorine in Water samples facilitated by in-situ pH control using interdigitated microelectrodes. Sens. Actuators B Chem..

[45] Wasiewska L., Seymour I., Patella B., Inguanta R., Burgess C., Duffy G., O’Riordan A. (2021). Reagent free electrochemical-based detection of silver ions at interdigitated microelectrodes using in-situ pH control. Sens. Actuators B-Chem..

[46] Seymour I., O’Sullivan B., Lovera P., Rohan J., O’Riordan A. (2021). Elimination of Oxygen Interference in the Electrochemical Detection of Monochloramine, Using In Situ pH Control at Interdigitated Electrodes. ACS Sens..

[47] Wahl A., Seymour I., Moore M., Lovera P., O’Riordan A., Rohan J. (2018). Diffusion profile simulations and enhanced iron sensing in generator-collector mode at interdigitated nanowire electrode arrays. Electrochim. Acta.

[48] Baracu A.M., Dinu Gugoasa L.A. (2021). Review—Recent Advances in Microfabrication, Design and Applications of Amperometric Sensors and Biosensors. J. Electrochem. Soc..

[49] O’Boyle S., Trodd W., Bradley C., Tierney D., Wilkes R., Longphuirt S.N., Smith J., Stephens A., Barry J., Maher P. (2019). Water Quality in Ireland 2013–2018.

[50] Wahl A., Barry S., Dawson K., MacHale J., Quinn A.J., O’Riordan A. (2013). Electroanalysis at Ultramicro and Nanoscale Electrodes: A Comparative Study. J. Electrochem. Soc..

[51] Burke L., Nugent P. (1997). The electrochemistry of gold: I the redox behaviour of the metal in aqueous media. Gold Bull..

