# Supplementary material for: Platinum-Based Interdigitated Micro-Electrode Arrays for Reagent-Free Detection of Copper
Source: Sensors (Basel). 2021 May 19;21(10):3544. doi: 10.3390/s21103544 (PMC8161293; doi:10.3390/s21103544)
Supplement: Supplementary file 1 [file sensors-21-03544-s001.zip › sensors-1188607-supplementary.pdf]

sensors

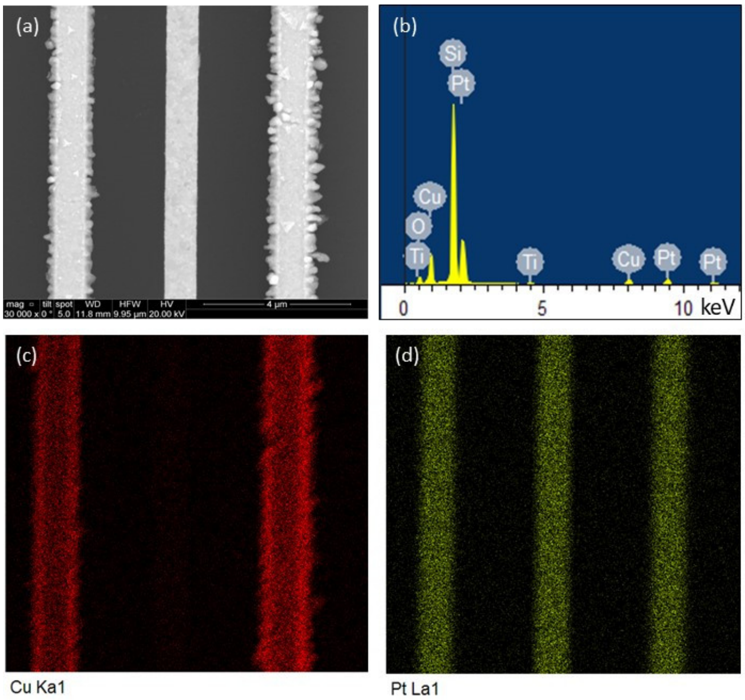

Figure 1. (**a**) SEM micrograph of the sensor after deposition of Cu from a 100µg/L solution (prior to stripping); The microbands on the left and on the right are part of the sensing comb while the microband in the middle is part of the protonator (**b**), EDX spectrum of the sample, showing Cu peaks at 1 and 8 keV; EDX mapping analysis showing (**c**) Cu and (**d**) Pt contributions.
